# Space positional and motion SRC effects: A comparison with the use of reaction time distribution analysis

**DOI:** 10.2478/v10053-008-0146-5

**Published:** 2013-12-31

**Authors:** Piotr Styrkowiec, Remigiusz Szczepanowski

**Affiliations:** 1 Institute of Psychology, University of Wroclaw, Poland; 2 University of Social Sciences and Humanities, Faculty in Wroclaw, Poland

**Keywords:** stimulus-response correspondence, Simon effect, space positional SRC effect, motion SRC effect, RTRT distribution analysis

## Abstract

The analysis of reaction time (RT) distributions has become a recognized standard in studies on the stimulus response correspondence (SRC) effect as it allows exploring how this effect changes as a function of response speed. In this study, we compared the spatial SRC effect (the classic Simon effect) with the motion SRC effect using RT distribution analysis. Four experiments were conducted, in which we manipulated factors of space position and motion for stimulus and response, in order to obtain a clear distinction between positional SRC and motion SRC. Results showed that these two types of SRC effects differ in their RT distribution functions as the space positional SRC effect showed a decreasing function, while the motion SRC showed an increasing function. This suggests that different types of codes underlie these two SRC effects. Potential mechanisms and processes are discussed.

## Introduction

The classicsta Simon effect refers to a situation in which participants are required to make a left or right manual response to a nonspatial feature of a visual stimulus (e.g., the colour of an object; see Simon, 1990, for a review). Motor responses tend to be faster and more accurate when the location of the stimulus corresponds with the location of the required response, despite the fact that the location of the target is irrelevant for performing the task (Simon & Rudell, 1967). This advantage is an example of a phenomenon known as *stimulus-response correspondence* (SRC). In particular, the Simon effect is a type of SRC that concerns *space positional correspondence*. Although the Simon effect has been widely studied in experimental psychology, the underlying factors of this effect are still a matter of ongoing debate (for a review, see Hommel, 2011; Proctor & Vu, 2006). With regard to the potential mechanisms responsible for the occurrence of the Simon effect, several models have been proposed: the two-route model (Kornblum, Hasbroucq, & Osman; 1990), the attention-shift account (Nicoletti & Umiltŕ, 1994), an updated version of the premotor theory of attention (Van der Lubbe, Abrahamse, & De Kleine, 2012), the event coding account (Hommel, Müssler, Aschersleben, & Prinz, 2001), the activation-suppression account (Ridderinkhof, 2002), or visuomotor activation (Wascher, Schatz, Kuder, & Verleger, 2001). However, none of these accounts seems to prevail, which comes as no surprise given that different research techniques and data analysis methods (e.g., of distributions) produced inconclusive results.

The same ambiguity applies to another example of an SRC phenomenon, the motion based SRC effect, for example, the *motion based type of the Simon effect*. Generally, this phenomenon refers to faster and more accurate responses when responses correspond spatially to the direction of the motion of the target object. This holds even if stimulus motion is irrelevant for the choice task at hand because participants respond to a visual feature other than motion (e.g., colour). This effect is sometimes treated as a variant of the classic Simon effect (e.g., Galashan, Wittfoth, Fehr, & Herrmann, 2008). In research on the motion-based Simon effect, spatial (i.e., positional) and motion cues are often confounded, causing difficulty in explaining the nature of this effect. Specifically, while having some motion feature, very often either the stimulus or the response is additionally located on the left or the right side (e.g., Nattkemper & Prinz, 2001; Wittfoth, Buck, Fahle, & Hermann, 2006). Thus, apart from the motion feature also the positional factor (i.e., the lateral location of the stimulus and/or the response) is involved. This positional factor may play a role in shaping the particular SRC effect. Most importantly, this might be the reason why the explanation of the nature of the motion based SRC effect lacks clarity. Two concurrent accounts attempt to explain the motion-based Simon effect: One is related to coding of the stimulus motion direction (see Figliozzi, Silvetti, Rubichi, & Doricchi, 2010), the other one refers to referential coding of stimulus motion (see Bosbach, Prinz, & Kerzel, 2004, 2005). What is important, these explanations are derived from theories on the classical space positional SRC phenomenon as they relate to the attentional shift and event coding accounts, respectively.

In the current study, we made a distinction between the classic Simon effect and its motion-based counterpart by claiming that both effects should be treated as separate phenomena. We expected to find differences between space-positional SRC and motion based SRC effects in terms of their temporal dynamics (i.e., how the magnitude of these effects changes as a function of the response speed). This was examined by comparing these effects by means of distribution analyses of reaction times (RTs).

Since the work of De Jong, Liang, and Lauber (1994), it has become a recognized standard in studies on the Simon and other correspondence effects to report not only RT differences between the corresponding and non-corresponding conditions, but also to include the analysis of RT distributions (Proctor, Miles, & Baroni, 2011). RT distributions for non-corresponding and corresponding trials for each participant are divided into quantiles, called bins. In most cases, the number of bins is between four and 10. The mean RT is calculated for each bin, and the difference between mean RTs for non corresponding and corresponding trial types is determined, which shows the magnitude of the SRC effect for each bin. Finally, the differences are plotted and analyzed as a function of bin and condition. When the size of the SRC effect is largest at the short RT quantile and then decreases, this is denoted as a decreasing distribution function. When the size of the SRC effect is small with the fastest responses (short RT bins) and becomes larger with slower responses (long RT bins), an increasing RT distribution function is observed. Proctor et al. (2011) reviewed studies using the RT distribution analysis of spatial correspondence effects and demonstrated that there is no consistency in the patterns of group distributions between different types of experiments on spatial (i.e., positional) correspondence effects. Nonetheless, the authors give arguments in favor of the claim that distribution functions indeed reflect the time course of processing underlying the Simon effect, a claim that was repeatedly questioned (Buetti & Kerzel, 2008; Roswarski & Proctor, 2003).

Proctor et al. (2011) point out that decreasing functions, in which the Simon effect is largest at the short RT bins and decreases across distribution, are mainly limited to the standard version of the Simon task (the classic Simon effect; e.g., Experiment 1 of De Jong et al., 1994; or Experiment 1 of Vallesi, Mapelli, Schiff, Amodio, & Umiltŕ, 2005). The decreasing magnitude of the Simon effect for slower responses is thought to support the idea of decay of automatically formed spatial response codes (Hommel, 1994). Other studies not listed by Proctor et al.’s review also provide evidence for the decreasing function (e.g., Van der Lubbe, Jakowski, & Verleger, 2005; Van der Lubbe & Verleger, 2002). On the other hand, increasing functions, when the SRC effect is small with the fastest responses and becomes larger with slower responses, are often obtained, for instance, when location information is provided by centrally located indicators (i.e., arrows; Pellicano, Lugli, Baroni, & Nicoletti, 2009) or when stimulus and response locations are arranged vertically (Experiment 1 of Proctor, Vu, & Nicoletti, 2003). Proctor and colleagues (2011) summarized this discrepancy as follows:

…although the patterns of these distribution functions for different versions of the Simon task are generally reliable, there is no current explanatory model that encompasses both when and why the Simon effect decreases in some cases and increases in others. (p. 263)

Despite the difficulties in explaining the Simon effect by the distribution analysis, the authors stress that it is a helpful tool and as such should be applied to other phenomena in which irrelevant stimulus information generates response competition.

There are several models that make an attempt to explain why the magnitude of the SRC effect changes as the function of the response speed. However, none of these accounts is capable of explaining why the one type of the SRC task leads to the decreasing RT distribution function whereas the other type of the SRC task elicits the increasing pattern of that function. In the present study, we refer to the visuomotor activation model proposed by Wascher et al. (2001), and to the activation-suppression model offered by Ridderinkhof (2002). Although these accounts might seem competing at first, they both offer a plausible explanation of the temporal dynamics of the SRC effect.

According to the model proposed by Wascher et al. (2001), the intra-hemispheric activation caused by a privileged visuomotor pathway accounts for the decrease of the Simon effect over time. Processing of a laterally presented stimulus causes increased activation in the hemisphere contralateral to the stimulus position. This, in turn, directly influences another activation which is related to response readiness with the effector that is under control of the same hemisphere (Wascher et al., 2001). Hence, the activation within the neuronal visuomotor pathway in the same hemisphere is responsible for faster and more accurate responding in the case of spatial correspondence between the stimulus and response. The model assumes that the visuomotor activation in corresponding trials relates to increased variance of RTs. Wascher et al. (2001) suggest that this increased variance of corresponding trials is a result of additional processes that are negatively time-consuming. This can be understood in terms of cognitive processing that accelerates subsequent processing. According to Zhang and Kornblum’s explanation (1997), larger variance of the RT distribution for corresponding than for non corresponding trials yields a larger difference between congruent and incongruent trials. As this activation dissipates, the variance of RTs for congruent trials and the difference between congruent and incongruent conditions decrease. This results in the negative slope of the distribution function (i.e., decreasing the Simon effect with increasing RTs). Additionally, in the first version of their theory, Wascher et al. claimed that visuomotor activation occurs only with left and right hand responses to horizontally arranged stimulus configurations. That is, visuomotor activation and the decreasing Simon effect functions should not be expected when stimuli and responses vary along the vertical dimension or when the response is unimanual. However, recent research seems to contradict Wascher and colleagues’ original hypothesis by showing that the decreasing distribution function may occur for unimanual responding (e.g., Buetti & Kerzel, 2008; Proctor & Vu, 2010; Wiegand & Wascher, 2007a). Moreover, there are studies demonstrating stable or increasing distribution functions even though stimuli were presented horizontally and responses were given with the left or right hands in a normal position (see Experiment 1 of Mapelli, Rusconi, & Umiltŕ, 2003; Experiment 1 of Gevers, Caessens, & Fias, 2005; and Experiment 1 of Gevers, Ratinckx, De Baene, & Fias, 2006). The above evidence contradicts the claims of Wascher et al.’s original model. This account was also criticized for being based heavily on physiology, and that experiments aimed at supporting this model were problematic in terms of their methodology as well as interpretation (see Roswarski & Proctor, 2003). This criticisms led Wascher’s group to modify their account (Wiegand & Wascher, 2007a, 2007b). Now, according to the modified view, it is not the spatioanatomical mapping itself that is responsible for the visuomotor activation but it may result from distinct mechanisms that rely on different spatial response codes. Direct activation of the corresponding response may be an effect of an overlap between spatial stimulus feature and one of the two distinct parameters of the motor code. Depending on the task these parameters represent either the spatial anatomical status of the effector or relative response location based on a cognitive mechanism (Wiegand & Wascher, 2007a). Yet, as Proctor et al. (2011) pointed out, this modification still requires more empirical support.

The activation-suppression model by Ridderinkhof (2002) delivers another explanation for the SRC effect, implicating that an activated response can be selectively inhibited. This account assumes that it takes some time for inhibition to build up, therefore, we observe an inhibitory effect after some time in response activation. Slower responses are thus affected in greater extent by selective inhibition than faster responses. Ridderinkhof’s account suggests that for faster responses on congruent trials the automatic activation processes (automatic route) facilitate the correct response, while on incongruent trials, these processes interfere with the correct response (Ridderinkhof, van den Wildenberg, Wijnen, & Burle, 2004). On the other hand, with slower responses, inhibition has time to develop, and can result in the reduction of activation of the incorrect response along the direct automatic route. Therefore, it is expected that correct responses for congruent trials can be less facilitated, whereas the correct responses to incongruent trials can be less delayed (Ridderinkhof et al., 2004). According to Ridderinkhof and colleagues, this will be reflected in the shape of the RT distribution function, which initially (i.e., for faster responses) will have a positive slope but with slower responses it will level off.

After a close inspection of the literature, we noted that there have been no reports so far on distribution analyses of the motion based SRC effect. Therefore, we will explore the time course of the classic Simon effect and the motion based SRC effect. By the former we mean an effect in a task in which participants respond with the left or right hand (by pressing a button) to a non-spatial feature of a static visual stimulus located either in the left or right visual field. A congruent lateral position of the stimulus and the response results in faster RTs compared to an incongruent position. By the latter effect we mean an effect in a task in which stimuli and responses share the feature of motion and only that feature determines SRC. We call that the *pure motion based SRC effect*. In this case, congruency of motion direction between stimulus and the response brings faster responses than the incongruent condition. In order to obtain this effect, the participant’s task should meet the following requirements:

1. The stimulus is displayed at central fixation and does not change its position.

2. Only the internal structure of the stimulus moves while the overall position of the stimulus remains the same.

3. To this end, a square object is formed by a group of vertical stripes, and the stripes move to the left or right, but never go beyond the borders of the square object.

4. Thus, the vertical lines within the square are displaced in either direction while the whole square remains stationary.

This technique allows for conveying motion information in the absence of a position shift (see Bosbach et al., 2004). In order to increase the dimensional overlap between stimulus motion and response, the participant performs joystick movements to the right or left in response to the color of the grating. The joystick is placed on the body midline and the participant operates it with his or her dominant hand (unimanual response setup). This setup allows for the pure motion based SRC effect to occur, because the spatial factor is limited or even excluded as the stimuli and responses are not located on either side.

The current study examined the time course of response activation within the spatial SRC effect and the motion based SRC effect. First, we replicated the standard visual left-right space positional correspondence task in order to obtain the classic Simon effect with the decreasing difference between non-corresponding and corresponding trials across RT bins. This task was set up as a control experiment for our studies. Then, we conducted three experiments, in which we progressively introduced a motion feature for the response and stimulus while limiting the spatial factor (side location) of the response and stimulus. This allowed us to eventually establish a task with the pure motion SRC. RT distribution functions were calculated in all four experiments. Similarly to De Jong et al. (1994), we reasoned that if the space positional SRC effect and the pure motion based SRC effect are different phenomena, this should be reflected in different shapes of the distribution functions for these effects. In reference to the Wiegand and Wascher (2007b) account, we suspected that the standard space positional SRC effect would lead to a decreasing RT distribution function while the motion based SRC effect would lead to an increasing distribution function as these two effects are probably based on different types of motor codes.

## Experiment 1

We used the standard visual left-right Simon task to examine the space positional SRC effect magnitude change as a function of response speed. Usually, in the typical visual Simon task, participants are required to make a left or right manual response (e.g., with the button press) to a non-spatial feature of the visual stimulus (e.g., colour), which is located in either the left or right visual field. In this setup, a lateral location of the stimulus and response is irrelevant for the task at hand, but this spatial factor is crucial for the occurrence of the SRC effect. This experiment was set to replicate findings reported in previous studies (see the Introduction section) and to serve as a control experiment. Thus we expected that a congruent lateral position of the stimulus and the response will result in faster responses compared to an incongruent condition. We also expected that this canonical setup will yield a decreasing RT distribution function.

### Method

#### Participants

Thirteen undergraduates (10 females, three males) took part in the experiment in exchange for a course credit. Participants were aged from 21 to 52 years (*M* = 28.9, *SD* = 8.3). All had normal or corrected-to-normal vision, and had no motor impairments as well as no former or current neurological disorders. All participants were right-handed as assessed with the Edinburgh Handedness Inventory (Oldfield, 1971) after the participant completed the task. Participants were naďve to the purpose of the experiment. All participants gave written informed consent before the experiment. The study was approved by the local ethical committee of the University of Social Sciences and Humanities, Faculty in Wroclaw.

#### Apparatus and stimuli

The experiment took place in a sound-attenuated and darkened room. Participants were seated in front of a 21-inch CRT monitor (with refresh rate of 100 Hz) controlled by a PC computer. Stimulus presentation and response recording was controlled by the Inquisit software system. An adjustable chin-rest was used to hold the participant’s head in a steady position. The distance between the eyes and the screen was approximately 60 cm. Responses were collected with the use of the buttons of two Saitek Aviator joysticks. The joysticks were located on the desk so that each participant could comfortably lay both hands on the desk while placing both hands on the joysticks’ bases. The middle buttons at the front of each joystick base were used; participants pressed the button on the left joystick base with their left thumbs, and the button on the right joystick base with their right thumbs. The distance from the left to the right joystick was approximately 50 cm. Each joystick was covered with a black box with specially-designed holes for hands so that participants were unable to see the joysticks, their hands, or their movements. This was introduced because we wanted to limit reciprocal visual information about hand movements as it might have an effect on stimulus-response interaction.

The stimuli used in the experiment were colour rectangular gratings formed by five vertical lines, which were presented on black background. Each stripe was ~0.5° wide and ~5° high. The space between the lines was of ~0.7° width. The area of the rectangular grating was around 5° × 5° in size. All stripes of the grating were either red or green. The rectangular grating was located in the middle of the vertical meridian of the screen, either on the left or on the right side. The distance between the centre of the screen and the centre of the rectangular grating was approximately ~7.5°. There were four types of stimuli: a red grating on the left, a red grating on the right, a green grating on the left, and a green grating on the right.

#### Procedure

Each trial begun with a black screen presented for 1,000 ms. Then, the colour grating was displayed for 200 ms along with a white fixation point shown in the centre of the screen. Next, another black screen was presented, and the participants indicated the colour of the vertical stripes by pressing the left or the right button with the left or the right thumb. The response period lasted for a maximum of 1,000 ms or until a response was recorded. Participants were asked to respond as fast and as accurate as possible. RTs were measured from stimulus onset. After each response, a feedback screen was presented for 1,000 ms,which informed the participant whether the answer was correct, incorrect, or whether there was no response. Then, a new trial started. Participants were instructed to maintain their eyes on the fixation dot that accompanied the stimulus during its brief presentation. The experiment consisted of two blocks so that in one block the left button press was assigned to the green colour and the right button press to the red colour, while in the other block, assignments of the colours to the buttons was reversed. Overall, there were 160 trials in each block with four types of colour gratings (40 red on the left side, 40 green on the right side, etc.) that were presented in random order. The number of congruent trials, in which the spatial stimulus location corresponded to response location (spatial congruence), was equal to the number of incongruent trials with no spatial correspondence between the stimulus and response. The order of blocks was randomized across participants. Before each block, participants were explicitly informed about the types of colour-response assignments. At the beginning of the experiment, participants were carefully instructed how to proceed with the task, and were given a short practice session.

To evaluate how the magnitude of the space positional correspondence effect changes as a function of response speed, a distributional analysis was conducted on RT data (De Jong et al., 1994; Proctor et al., 2011). Only RTs for correct responses were included. Trials with RTs faster than 100 ms (< 0.5%) were excluded from the analysis. For each participant RT data were ordered and ranked in respect to five bins partitioned into quantiles separately for congruent and incongruent condition. Each bin had a range of 20 percentiles. Low bins related to faster responses whereas higher bins related to slower responses. Next, the mean RT was calculated for each bin. Subsequently, for each bin the difference between mean RTs for non corresponding and corresponding conditions were determined. Finally, these differences were plotted and analyzed as a function of bin and condition.

All statistical analyses were conducted using IBM SPSS software. To correct for violations of the sphericity assumption in all ANOVAs, the Greenhouse-Geisser correction was used.

### Results

The mean RTs for correct responses were subjected to a two-way repeated-measures analysis of variance (ANOVA) with the independent variables of Bin (with five levels: from the first to the fifth bin) and Space Positional Congruency Between Stimulus and Response (congruent vs. incongruent). ANOVA revealed the main effect of space positional congruency, *F*(1, 12) = 7.23, *p* = .02, with faster RTs when the stimulus location corresponded to the response location (472 ms) as compared to the condition without such correspondence (499 ms; see Table 1). Trivially, the main effect of bin was observed, *F*(4, 48) = 300.5, *p* < .001.

**Table 1.** Summary of RTRT Results for Different SRCSRC Effects for Experiments 1-4

	Mean RTs for congruent trials	Mean RTsfor incongruent trials	Size of the SRC effect	Means of five RT distribution quantiles (bins)	Magnitudes of SRC effect for five bins
Type of stimulus-response correspondence					
Stimulus position –Response position. Experiment 1	472(15)	499(16)	27	365, 428, 473, 525, 638	32, 45, 37, 23, -3
Stimulus position – Response movement direction. Experiment 2	532(10)	564(11)	32	434, 493, 537, 589, 690	41, 46, 44, 33, 9
Stimulus position – Response movement direction. Experiment 3	532(11)	577(12)	45	432, 493, 538, 595, 714	51, 57, 55, 44, 15
Stimulus motion – Response movement direction. Experiment 4	488(16)	504(19)	16	389, 442, 482, 527, 641	5, 10, 16, 22, 26

*Note*. RT = reaction time (in milliseconds). SCR = stimulus response correspondence. Standard deviations in parentheses.

**Figure 1.**
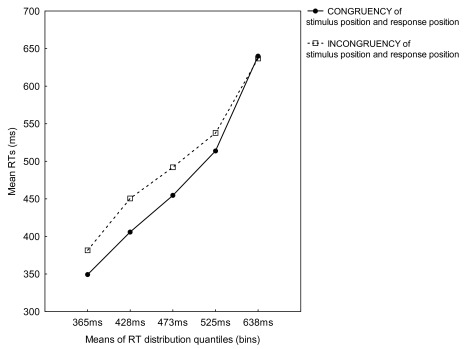
Mean reaction times (RTs) for congruent and incongruent trials for five RT distribution quantiles (bins) from Experiment 1.

Of most interest, a significant interaction between Bin and Space Positional SRC, *F*(4, 48) = 9.06, *p* < .001, showed that for all bins, mean RTs for the congruent condition were faster than for the incongruent condition, and that in the congruent condition, the RT increase was steeper than in the incongruent condition (see Figure 1).

**Figure 5.**
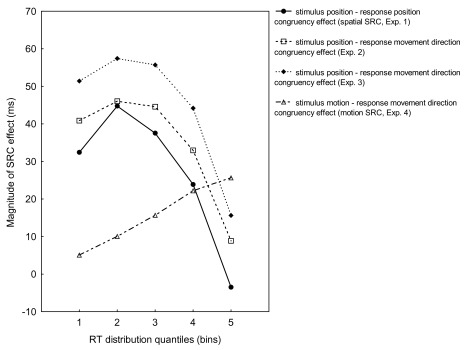
Reaction time (RT) distribution functions of stimulus-response correspondence effects from Experiments 1, 2, 3, and 4. SRC = stimulus response correspondence.

For each participant, the mean congruent RTs were subtracted from the incongruent mean RTs in order to obtain the plot of the RT distribution function (see Figure 5, solid line). The resulting pattern of the RT distribution function for the positional SRC effect showed an inverted j-shaped curve with the decreasing course. In particular, the space positional correspondence effect function peaked at about 45 ms at the second bin, and then decreased to about -3 ms at the fifth bin. The exact values of the SRC effect were 32, 45, 37, 23, and -3 ms from the shortest to the longest bin. To further examine the slope of the RT distributions, the size of the space positional SRC effect for all bins was subjected to a linear regression analysis. The regression analysis indicated that this effect decreased as a function of the bin, β = -0.30, *t*(64) = -2.53, *p* = .014. In fact, the curvilinear pattern of the space positional correspondence effect function was confirmed by the quadratic model of regression, β_1_ = 0.90, *t*(62) = 1.49, *p* = .14, β_2_ = -1.23, *t*(62) = -2.03,*p* = .046.

The analysis of accuracy did not show significant differences in the results: Participants reached the level of 94% of correct answers in the space positional congruency condition and 90% in the incongruent condition (*p* = .30).

### Discussion

Consistent with previous research (see Simon, 1990; see also Hommel, 2011), our control Simon task showed a robust space positional SRC effect. This effect was not present in our accuracy measures. In addition, the results from the RT distribution analysis indicated that the positional SRC effect decreased as RTs increased, which is also consistent with previous findings on temporal activation properties for the Simon effect (see Proctor et al., 2011, for a review). This decrease of positional SRC effect magnitude over time conforms to the idea of the decay of automatically formed spatial response codes (Hommel, 1994). The resulting pattern of the RT distribution function is also in line with the visuomotor activation hypothesis (Wascher et al., 2001), which assumes that visuomotor activation that results from distinct motor codes dissipates over time which leads to a decrease of the congruency effect with longer responses. A closer investigation of the Simon effect function demonstrates that the space positional SRC effect changed nonlinearly over time. Clearly, the function has a curvilinear shape with a maximum between the first and the second bin, until it displays a typical decreasing character. Such a pattern of results is similar to outcomes reported by Davranche and McMorris (2009), who had participants perform the Simon task with thumbs. These researchers have explained the curvilinear shape of the distribution function with reference to the activation-suppression model. Interestingly, our plot for the RT distribution function also appears to corroborate the activation-suppression model by Ridderinkhof (2002). Because suppression of automatic activation needs some time to develop, initially the spatial dimensional overlap causes response activation that produces an increase of the correspondence effect magnitude. After a while, when suppression comes into play, activation becomes progressively inhibited. The initial increase of the effect size may be stopped and a decrease is more likely to begin. This effect is reflected in the inverted j-shaped curve of the RT distribution function. Although both models (i.e., the visuomotor activation and the activation-suppression accounts) predict the decrease of the spatial SRC effect in Simon tasks, it seems that the activation-suppression model fits better with the curvilinear effect function.

## Experiment 2

The task used in this experiment was designed to introduce a motion feature of the response, while the location factor of the response was limited. In this task, participants responded to the colour of the stimulus located on the left or right side by leftward or rightward joystick stylus movements. Although there was a movement feature involved in this task (which was absent in the task from Experiment 1), the SRC had a position-referential character, that is, the stimulus was located on either side and the response was performed in either direction, in reference to the stimulus position. Importantly, as the joystick was positioned in the centre, on the body midline, and was operated with the participant’s dominant hand (the unimanual response setup), the positional aspect of the response was limited. Although there was a factor of motion, we assumed that the SRC effect in this experiment should be rather a spatial phenomenon. We expected thus that the magnitude of the effect associated with correspondence between stimulus location and response movement (in the direction of the stimulus location) would decrease with longer RTs. Although Wascher et al. (2001) suggested that with the unimanual responses the RT distribution function should be stable or increase, we assumed that in our experiment this function should decrease because of the dominant role of the position-referential (i.e., spatial) factor.

### Method

#### Participants

Thirteen students (nine females, four males) from the same population as in Experiment 1 took part in the experiment in exchange for course credits. None of the volunteers participated in the previous experiment. Their age ranged from 19 to 32 years (*M* = 22.7, *SD* = 4.9).All had normal or corrected-to-normal vision, and had no motor impairments and neurological disorders. All participants were right-handed as assessed with the Edinburgh Handedness Inventory (Oldfield, 1971).

#### Apparatus and stimuli

Experiment 2 was carried out in the same experimental setup as Experiment 1, with the following modifications.

In this experiment, responses were collected with one joystick (Saitec Aviator), positioned centrally with respect to the body midline. The joystick’s position allowed participants to comfortably lay their hand on the desk. Participants held the joystick with their dominant hand. Before the procedure, each participant was asked to imagine with which hand she or he would prefer to grasp a tennis racket. The hand to hold the joystick was selected accordingly. The black box was also used to cover the hand for the same purpose as in Experiment 1.

The stimuli were exactly the same as in Experiment 1.

#### Procedure

The stimuli were presented in the same fashion as in the previous experiment. The only modification within the procedure regarded responding. Participants were asked to indicate the colour of a stimulus by pushing the joystick either to the left or right. Participants were told to shift the joystick to its original upright position as quickly as possible right after each response. RTs were measured from stimulus onset. The beginning of the leftward or rightward joystick movement was treated as the start of the reaction. There were two blocks with 160 trials each. In one block, the leftward movement by joystick tilting was assigned to the red lines, whereas the rightward movement was assigned to the green lines; the colour assignment in the other block was reversed. In all other respects this experiment was identical to the previous experiment.

### Results

Mean RTs for correct responses were subjected to a two-way repeated measures ANOVA with the independent variables of Bin and Congruency Between Stimulus Location and Response Movement Direction (congruent vs. incongruent). ANOVA revealed the main effect of congruency between the stimulus location and response movement direction, *F*(1, 12) = 31.42, *p* < .001, with faster RTs for congruent (532 ms) as compared to incongruent trials (564 ms; see Table 1). Trivially, a main effect of bin was observed, *F*(4, 48) = 347.3, *p* < .001.

**Figure 2.**
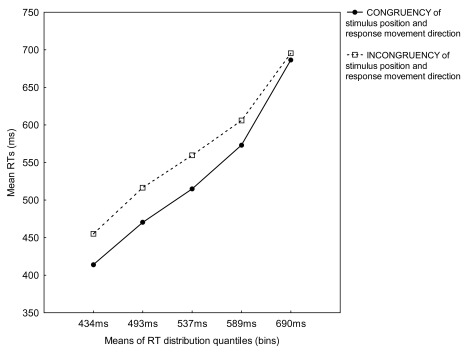
Mean reaction times (RTs) for congruent and incongruent trials for five RT distribution quantiles (bins) from Experiment 2.

Of most interest, a significant interaction between Bin and Congruency, *F*(4, 48) = 16.89, *p* < .001, showed that for all bins mean RTs for the congruent condition were faster than for the incongruent condition, and that the RTs increase was steeper for the congruent than for the incongruent condition (see Figure 2).

For each participant, mean RTs for congruent trials were subtracted from mean RTs for incongruent trials to generate a group RT distribution function (see Figure 5, dashed line). The resulting pattern of the RT distribution function for the position-referential correspondence effect showed an inverted j-shaped curve with the decreasing course. In particular, the SRC effect function peaked at about 46 ms at the second bin, and then decreased to about 9 ms at the fifth bin (the effects were 41, 46, 44, 33, and 9 ms from the shortest to the longest bin). To further examine the slope of the RT distribution function, the sizes of the SRC effects for all bins were subjected to the linear regression analysis, which revealed that this effect decreased as a function of the bin, β = -3.91, *t*(64) = -3.37, *p* = .001. In fact, the curvilinear pattern of the SRC effect function was confirmed by the quadratic model of regression, β_1_ = 1.1, *t*(62) = 1.92, *p* = .059, β_2_ = -1.5, *t*(62) = -2.66, *p* = .01.

The analysis of accuracy did not show significant differences, indicating that participants reached the level of 96% of correct answers in the congruent S-R condition, and 94.6% in the incongruent condition (*p* = .35).

### Discussion

Experiment 2 demonstrated a robust congruency effect between the spatial location of the stimulus and the response movement toward stimulus position, which is another kind of the SRC effect. This SRC effect may be treated as position-referential in its character, because space positional factors play an important role here: The stimulus is laterally located and the response is performed toward the side of that stimulus (i.e., in reference to its position). Thus, it suggests that the positional factor may be significant in mechanisms of stimulus-response matching. It was shown via the distribution analysis that the congruency effect between stimulus location and response movement toward a lateralized position decreased with longer RTs. This result is consistent with findings of the SRC studies employing unimanual responses, which contradict the claim proposed by Wascher et al. (2001) that for this response condition, an increasing distribution function should be found (e.g., Buetti & Kerzel, 2008; Proctor & Vu, 2010). In our study, responses were given with the dominant hand and the size of the SRC effect was decreasing and not increasing over time. Thus, the result of our experiment adds to the argument against spatioanatomical mapping, originally postulated by Wascher et al. (2001) in their visuomotor activation account of the SRC phenomenon. Additionally, the RT distribution function of the correspondence effect from Experiment 2 has the shape of the j-inverted curve, and it seems that, as in the previous experiment, it is the Ridderinkhof (2002) activation-suppression model that explains this pattern of results more comprehensively.

## Experiment 3

The difference between this and the previous experiment concerned the feature of motion that was added to the characteristics of a stimulus. In Experiment 3, stimuli apart from being laterally positioned possessed also the feature of motion, and the response had only the feature of motion. In the previous experiment, the stimulus had only a positional feature, and the response had only the motion feature. This motion feature of the stimulus in Experiment 3 was obtained by employing a coloured sine-wave grating described in the Introduction section. In this version of the task, the stationary-moving stimulus was presented on either the left or the right side. The stationary-moving stimulus had two distinct space positional and motion features: left versus right location and leftward versus rightward movement of the vertical lines within the stimulus, respectively. The whole stimulus was stationary and although it had a feature of motion, the stimulus was not changing its spatial position. The responses were given by pushing the joystick’s stylus in the left or right direction using the dominant hand (the unimanual response setup). Two types of SRC can be distinguished in this task: (a) correspondence between stimulus position and the direction of response movement, and (b) correspondence between the within-stimulus motion direction and the direction of the response movement. We called the first one the position-referential SRC and the other one the motion-directional SRC. Furthermore, we assumed that the referential SRC is more spatial in nature whereas the directional SRC is a more motion-based phenomenon (i.e., other types of representational codes may play a role in these phenomena). As such, we claimed that in this experiment, these two kinds of the SRC effect should occur independently from each other (there should be no interaction between these two effects). Additionally, the RT distribution functions for these two effects should differ, which is in line with the rationale behind experiments reported by De Jong et al. (1994). To some extent, similar work was done by Nattkemper and Prinz (2001) and by Bosbach, Prinz, and Kerzel (2005), though in their studies, the stimulus motion feature was related to its position shift, that is, the stimulus was moving as a whole. Moreover, in these studies, the SRC effects were not compared using distribution analyses.

### Method

#### Participants

Thirteen students (eight females, five males) from the same population as in Experiment 1 took part in exchange for course credits. None of the volunteers participated in Experiment 1 or 2. Their age ranged from 19 to 31 years (*M* = 21.92, *SD* = 3.4). All had normal or corrected-to-normal vision, and had no motor impairments and neurological disorders. All participants but one (ambidextrous) were right-handed as assessed with the Edinburgh Handedness Inventory (Oldfield, 1971).

#### Apparatus and stimuli

Experiment 3 was carried out in the experimental setup used for Experiments 1 and 2. Responses were furnished and recorded in the same fashion as in Experiment 2. The experiment used a rectangular grating made up of red or green coloured vertical stripes of a similar size to those used in Experiments 1 and 2. In each trial, the stimulus was located either on the left or on the right side of the screen (in the same manner as in the previous experiments). However, Experiment 3was modified in such a manner that all vertical stripes of the grating were displaced (shifted) in one direction (left or right) with a speed of ~18°/s within the range of the grating area. When one stripe reached the edge of the rectangular area, it disappeared there, and a new stripe appeared on the opposite edge, giving the impression of constant motion inside the rectangular aperture. The coloured moving lines were displayed for 200 ms what means that during the stimulus presentation each line travelled ~3.6°. That in turn means that about three lines left subsequently the grating at one side, and three new lines appeared subsequently on the other side of the grating. The task employed two co-lours of vertical lines (red and green), two locations of the grating (left or right), and two directions of line motion (leftward or rightward), yielding eight types of stimuli.

#### Procedure

The same procedure was used here as in Experiment 2 with one exception, that is, the number of trials within each block was doubled (320 trials), because there were two types of SRC. The percentage of S-R position-referential congruent and incongruent trials was equal to the percentage of motion-directional S-R congruent and incongruent trials.

### Results

First, we conducted a two-way repeated-measures ANOVA with the independent variables of Congruency Between Stimulus Location and Response Movement Direction (congruent vs. incongruent), and Congruency Between the Direction of Within Stimulus Motion and Response Movement (congruent vs. incongruent). A main effect of congruency between stimulus location and response movement direction was observed, *F*(1, 12) = 47.33, *p* < .001, indicating the position-referential SRC effect. That is, correspondence between the location of the stimulus and the direction of response movement led to faster RTs (532 ms) as compared to the lack of such correspondence (577 ms; see Table 1). It turned out that neither the main effect of motion-directional SRC (i.e., congruency between the direction of within stimulus motion and response movement), *F*(1, 12) = 1.76, *p* = .21, nor the interaction between these two effects were significant, *F*(1, 12) < 0.001, *p* = .99.

**Figure 3.**
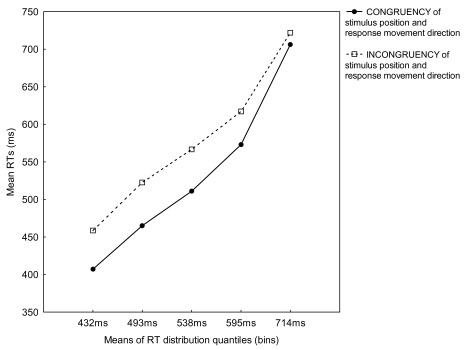
Mean reaction times (RTs) for congruent and incongruent trials for five RT distribution quantiles (bins) from Experiment 3.

Furthermore, as only the position-referential SRC effect was significant (i.e., congruency between stimulus location and the direction of response movement), we conducted another analysis of variance with variables of Bin (1 to 5) and Congruency Between Stimulus Position and Response Movement Direction (congruent vs. incongruent). Trivially, there was a main effect of bin, *F*(4, 48) = 437.84, *p* < .001. We also found a main effect of congruency, *F*(1, 12) = 47.17, *p* < .001. The ANOVA also revealed a significant interaction between Bin and Congruency, *F*(4, 48) = 19.19, *p* < .001, showing that for all bins, mean RTs for the congruent condition were faster than for the incongruent condition, and that the RT increase in the congruent condition was steeper than in the incongruent condition (see Figure 3).

Next, we generated the group RT distribution function (see Figure 5, dotted line). Its resulting pattern for the SRC effect showed again an inverted j-shaped curve with the decreasing course. In particular, the distribution function peaked at about 57 ms at the second bin, and then decreased to about 15 ms at the fifth bin. The exact values of the effect from the shortest to the longest bin were 51, 57, 55, 44, and 15 ms. To further examine the slope of the RT distributions, the sizes of the SRC effect were subjected to the linear regression analysis, which revealed that this effect decreased as a function of the bin, β = -4,0, *t*(64) = -3.48, *p* = .001. In fact, the curvilinear pattern of the position-referential correspondence effect function was confirmed by the quadratic model of regression, β_1_ = 1.20, *t*(62) = 2.12, *p* = .04; β_2_ = -1.63, *t*(62) = -2.89, *p* = .005.Although the results of the main ANOVA did not reveal the effect of the motion-directional SRC, we performed the RTs distribution analysis for this effect. This analysis also brought insignificant results (*p* = .82). It turned out that for each bin there were similar and very small (approximately 3 ms) differences between congruent and incongruent conditions. This stable RTs distribution function confirms that the motion-directional SRC effect was absent in that experiment.

The analysis of accuracy showed no significant differences for both types of SRC. Participants reached the level of 96% of correct answers for *position-referential SRC* and 93% when there was not such congruency (*p* = .097). For *motion-directional SRC*, response accuracy was at the level of 95% for the congruent condition and 94% for the incongruent condition (*p* = .12).

### Discussion

Experiment 3 provided conditions that enabled eliciting two types of the SRC effects. The first SRC was related to congruency between stimulus location and the direction of response movement. In our view, in this SRC, called by us the position-referential SRC, the spatial factor plays an important role. The second SRC was based on congruency between the direction of within-stimulus motion and the direction of response movement. We named this effect the motion-directional SRC, and within this phenomenon the motion factor is more important for the stimulus-response link.

Both effects were hypothesized as independent and were therefore not expected to interact. Although there was no interaction between these two effects, it was because only one of them occurred, namely, the effect of congruency between stimulus location and the direction of response movement (position-referential). The other effect (motion-directional), surprisingly, turned out to be non-significant.

The distribution analysis showed that the position-referential SRC effect tended to decrease with longer RTs, although initially there was a little increase of the size of this effect. These findings fit with the results obtained in Experiment 2. Moreover, we also found no significant effect of congruency between the direction of stimulus motion and the direction of response movement (motion-directional). This may suggest that in such conditions as those used in our experiment, where these two kinds of SRC are combined, the space positional factor probably dominates and determines which stimulus response congruency is privileged. Thus the position-referential effect occurred at the cost of the dissipation of the motion-directional effect. When the space positional factor is involved (as in this case due to a particular stimulus location in the condition of congruency between stimulus position and response movement direction), it is rather the position referential coding (see Bosbach et al., 2004; Nattkemper & Prinz, 2001) that is responsible for processing of the stimulus-response connection.

## Experiment 4

The final experiment involved the pure motion SRC, while the space positional SRC was excluded. This manipulation was achieved by placing the stationary-moving stimulus in the centre of the screen in order to avoid its presentation on either side. The participants were forced to respond in the unimanual fashion by using the joystick located centrally on the body midline. Here, we expected only the effect of congruency between within-stimulus motion direction and the direction of response movement, because no spatial location was involved in this task. It was hypothesized that the motion SRC effect should have a stable or increasing RT distribution function. We expected such RT distribution function because we suspected that this SRC effect is based on different type of codes than the space positional SRC effect, in accordance with claims of the Wiegand and Wascher account (2007b). In line with their account, we claim that position-referential codes are distinct to motion codes.

### Method

#### Participants

Thirteen students (seven females, six males) from the same population as in the previous experiments took part in the study in exchange for course credits. None of the volunteers participated in previous experiments. Their age ranged from 20 to 43 years (*M* = 26.7, *SD* = 7.19). All had normal or corrected-to-normal vision, and had no motor impairments and neurological disorders. Handedness of participants was assessed with the Edinburgh Handedness Inventory (Oldfield, 1971), and it appeared that 10 participants were right-handed, and three participants were left-handed.

#### Apparatus and stimuli

Experiment 4 employed the same materials as Experiment 3 with only one exception, namely, in this version of the task, we used a coloured sine-wave grating that was always presented in the centre of the screen.

#### Procedure

The procedure was the same as in Experiment 3, however, there was one modification concerning the number of trials within each block (160 trials). The percentage of trials with congruency between within-stimulus motion and response movement was equal to the percentage of incongruent trials.

### Results

RTs for correct answers were subjected to a two-way repeated-measures ANOVA with two variables: Bin (1 to 5) and Congruency Between Stimulus Motion and Response Movement (congruent vs. incongruent). There was a main effect of congruency between stimulus motion and response movement, *F*(1, 12) = 12.8, *p* = .004, with faster RTs when motion SRC was present (488 ms) as compared to the situation without such congruency (504 ms; see Table 1). Trivially, there was also a main effect of bin, *F*(4, 48) = 332.42, *p* < .001.

**Figure 4.**
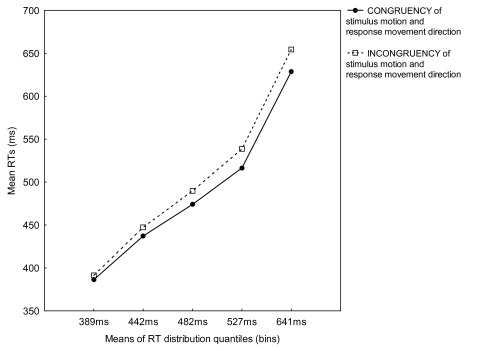
Mean reaction times (RTs) for congruent and incongruent trials for five RT distribution quantiles (bins) from Experiment 4.

Of most interest, a significant interaction between Bin and Congruency, *F*(4, 48) = 4.68, *p* = .031, showed that for all bins, mean RTs for the congruent condition were faster than for the incongruent condition. This time, however, the increase of mean RTs for incongruent trials compared to the congruent condition was steeper for all bins (see Figure 4).

Finally, we plotted the group RT distribution function (see Figure 5, dotted-dashed line). The motion SRC effect was 5, 10, 16, 22, and 26 ms from the first to the fifth bin, respectively. This suggested that the congruency effect was gradually increasing with longer RTs. In fact, the linear increase of the magnitude of the motion SRC effect as RT increased was also confirmed by the linear regression analysis, β = 0.36, *t*(64) = 3.07, *p* = .003.

Analysis of accuracy measures showed a simple effect of congruency with better accuracy for congruent (97%) than for incongruent (95%) trials; *t*(12) = 3.60, *p* = .004.

### Discussion

In Experiment 4, a robust motion SRC effect was obtained as RTs and accuracy were affected by congruency between stimulus motion and response movement. That is, faster and more accurate responses were observed when stimulus motion and the direction of the hand movement were congruent as compared to the incongruent condition. Importantly, the motion SRC effect was observed even though stimulus motion was task irrelevant (i.e., performance of the task did not depend in any respect on taking into account in what direction the lines were moving). It seems that our experiment provides a useful and valid technique for eliciting another SRC effect, namely, the motion SRC effect which bases solely on consistency between stimulus and response movement.

It should be stressed that our version of the task excluded the positional factor as neither the stimulus nor the response was located on the lateral side. Although this motion SRC effect is not large in size (16 ms) as compared to the space positional SRC effect (27 ms) from control Experiment 1, it seems that the results obtained in Experiment 4 are robust.

Of particular interest is how the motion SRC effect changes as a function of the response speed. The distribution analysis demonstrated the gradual and pronounced increase of the difference between the motion incongruent and congruent trials over time, which is reflected in a positive slope of the difference plot. This finding is consistent with the original visuomotor activation model, which implicates a flat or increasing distribution function of the SRC effect when the response is given unimanually. Although this claim was undermined by results from various studies on SRC (e.g., Buetti & Kerzel, 2008; Proctor & Vu, 2010; Wiegand & Wascher, 2007b), one important issue needs to be discussed here. It might be the confounding role of the spatial factor (in the form of lateral stimulus location) that is responsible for the occurrence of the decreasing distribution function in studies with unimanual responses. To some extent the claim about the confounding role of laterally located stimuli with one hand responses receives support in the results of the direction selection condition in Experiment 1 by Buetti and Kerzel (2008). In their experiment, participants responded with one hand to horizontally or vertically organized stimuli. For the horizontal direction condition, the stimulus could be located to the left or to the right of the fixation point, whereas in the case of a vertical direction condition, the stimulus was placed above or below the fixation point, thus in the midline. A decreasing SRC effect function was obtained for the horizontal stimulus and response location, whereas a flat (i.e., not decreasing) function occurred when the stimuli and responses were arranged vertically. Thus, when the stimulus was not located on the left or the right side, there was no decreasing distribution function. The spatial factor in the form of the lateral location of the stimulus may thus determine the decreasing function.

Nonetheless, the most important finding revealed in this experiment is that the pure motion SRC effect occurs (i.e., it is a real phenomenon) and the size of this effect increases over time. Although it is possible that the spatial factor (stimulus location) is a confounding variable that may play a role in determining whether the distribution function is decreasing or increasing, we think that it is something else that determines why the space positional SRC effect and the motion SRC effect have different distribution functions. We tentatively suggest that the differences in the distribution functions for the space positional and motion SRC effects reflect two qualitatively different types of codes that underlie the occurrence of these phenomena, which we will discuss in turn.

## General discussion

In the current study, we compared the space positional SRC effect and the motion SRC effect using RT distribution analysis. Experiment 1 showed a robust space positional SRC effect (i.e., the classic Simon effect). Positional incongruence between the stimulus and response, which was task irrelevant, resulted in slower RTs compared to the condition in which the location of the stimulus and response corresponded. Results of our replication of the Simon task are consistent with a considerable body of evidence (see Hommel, 2011; Proctor & Vu, 2006). Also, the distribution analysis conducted on RTs obtained in Experiment 1 showed results consistent with findings from other studies. Generally, this analysis shows that the Simon effect decreased over time. That is, the differences between corresponding and non-corresponding trials are larger for the fast RT bins than for the slow RT bins. This result was repeatedly observed in other studies (see Proctor et al., 2011). There are two non-mutually exclusive theories that account for the decreasing function of the Simon effect, namely, the visuomotor activation hypothesis of Wascher et al. (2001) and the activation-suppression hypothesis of Ridderinkhof (2002). Inspection of the j-shaped curvilinear RT distribution function of the Simon effect magnitude from Experiment 1 indicates that it is rather Ridderinkhof’s model (see the Introduction section) that better accounts for this shape of RT distribution function of the spatial SRC effect.

In Experiment 2, another kind of the SRC effect was demonstrated: Congruency between stimulus position and the direction of the unimanual response movement resulted in faster RTs compared to the condition without such congruency. The analysis of the RT distribution function of this SRC effect revealed a decreasing tendency similar to that obtained in Experiment 1. It seems that the shape of the RT distribution function of this effect is also explained by the Ridderinkhof account.

Experiment 3 used experimental conditions where two types of stimulus response congruency were possible. In particular, the direction of the unimanual response movement could be congruent with stimulus location and/or stimulus motion direction, while both features of the stimulus were task irrelevant. It turned out that only one SRC effect occurred: the effect related to congruency between stimulus location and the direction of response movement (i.e., the same SRC effect as in Experiment 2). The RT distribution function of this effect was also decreasing as in the previous experiment. Interestingly, the effect of congruency between stimulus motion and response movement (the motion SRC effect) was absent.

It seems that when there is more than one type of SRC in a single task, one dominates and overrides the other. Additionally, the dominating SRC is based on positional features of the stimulus (i.e., its lateral location). Thus, we suggest that position coding may play an important role in the processes underlying stimulus-response congruency.

The motion-based SRC effect was obtained in the last experiment when the space positional factor was excluded, since stimulus and response positions were not lateralized. This manipulation allowed the pure motion SRC effect to occur. This effect was obtained in conditions where there was congruency or incongruence between the stimulus and response motion features present. The task irrelevant direction of stimulus motion congruent with the unimanual response movement direction resulted in faster and more accurate responses as compared to motion incongruence. This effect represents another example of the SRC phenomenon. In fact, the pure motion SRC effect is distinct from another SRC related phenomenon, namely, the motion-based Simon effect. Since the latter is a combination of the positional and motion factors, it is difficult to understand the nature of this particular effect. The manipulation employed in our experiment allowed separating these factors and helped to understand better the characteristics of the SRC phenomena.

The current research provides a paradigm for eliciting the SRC effects linked to motion congruency that uses only consistency between movements of the stimulus and response, but does not involve space positional confounds. In addition, the motion SRC effect elicited by our task underlines the importance of both aspects of SRC phenomena, such as movements of both the stimulus and the response. These findings support Hommel’s (2011) view that either a stimulus or response may have the same impact on S-R interconnections. In his review, Hommel (2011) stresses that many studies focus mainly on perceptual processing linked with attention or spatial coding, while they tend to ignore some important aspects of the response and motor information processing. Our motion SRC design resolved this methodological issue by treating these factors as equally important.

To our knowledge, there are no reports that have taken into account the RT distribution analysis for the motion SRC effect like the one elicited in Experiment 4, indicating in fact the opposite result as compared to the distribution function in the standard Simon effect (i.e., space positional SRC), as found in Experiment 1. In particular, the RT distribution function of the motion SRC effect had a linear shape with a positive slope indicating that differences between stimulus-response motion congruent and incongruent trials increased with longer RTs. In our view, this fact of different distribution functions of the space positional and motion SRC effects reveals that they are different in nature, which supports our hypothesis based on reasoning similar to that of De Jong et al. (1994), stating that if two effects operate using different processes, it should be reflected in different distribution functions.

In our view, the different characteristics of the time course of the spatial SRC and motion SRC effects reflect their different underlying mechanisms. For instance, Wiegand and Wascher (2005, 2007a, 2007b) suggest that there are two types of mechanisms that are involved in the SRC phenomena. The first mechanism is the visuomotor automatic activation related to spatial anatomical mapping, which is responsible for the occurrence of the transient spatial SRC effect (i.e., the classic Simon effect). Sustained SRC effects (i.e., effects for which stable or increasing distribution functions are found) involve more cognitive mechanisms, as Wiegand and Wascher’s assumption posits. We state that the motion SRC effect from Experiment 4 employs more complex cognitive mechanisms as compared to the visuomotor activation involved in the space positional SRC effect. The motion feature provides more complex information about the stimulus than the mere positional information, because encoding object motion requires processing of space-time information (see Bruce, Green, & Georgeson 2003). Also, performance of the leftward or rightward joystick movements seems to be a more complex motor activity engaging more cognitive processes than a mere button press. This is supported by the comparison of mean RTs for unimanual responses performed by right-handed participants in Experiment 4 (joystick movements) and mean RTs for responses given by right-handed participants with their right-hands in Experiment 1 (button presses). In the first case, they amounted to 498 ms, while in the latter to 479 ms. Also, mean RTs for responses performed by right-handed participants using a joystick in Experiment 2, where stimuli were the same as in Experiment 1, were longer (548 ms) than mean RTs for button press responses. These data give support to the idea that moving a joystick is a more complex motor activity (and thus requires more information processing) than a simple button press. Altogether, this suggests that more cognitive mechanisms are engaged in the motion SRC effect as compared to the space positional SRC, and as Wiegand and Wascher (2005) suggest, these cognitive mechanisms are responsible for an increasing RT distribution function of the motion SRC effect.

There is also another possible factor that might be responsible for the occurrence of larger motion SRC effect with slower than with faster responses. As *motion* is defined as a change of location across time, it is logical that it takes time to perceive any motion. Very quick responses to the colour may cause that motion is not perceived and processed. Only with slower responses motion of the stimulus is detected. Hence, the correspondence effect between stimulus motion and the response movement is able to arise at a later stage when the motion is processed. Although the idea of time competition between different types of codes received some support (see Ansorge, 2003), further studies will help to verify this claim.

In conclusion, the current study seems to support the idea that the space positional SRC effect (the classic Simon effect) and the motion SRC effect represent two distinct SRC phenomena. This observation is supported by different shapes of the RT distribution functions, suggesting involvement of two qualitatively different mechanisms.
